# Multi-Trajectories of Conduct Problems, Hyperactivity/Inattention, and Peer Problems Across Childhood: Results from the Growing Up in Scotland Birth Cohort

**DOI:** 10.1007/s10802-022-00956-8

**Published:** 2022-08-03

**Authors:** María Francisca Morales, Angus MacBeth, Samantha Swartzman, Lisa-Christine Girard

**Affiliations:** Department of Clinical Psychology, School of Health in Social Science, The University of Edinburgh, Medical School (Doorway 6), Teviot Place, Edinburgh, EH8 9AG UK

**Keywords:** Conduct problems, Hyperactivity/inattention, Peer problems, Group-based multi-trajectory, Growing up in Scotland

## Abstract

Using a person-centred approach, this study inspected multi-trajectories of conduct problems, hyperactivity/inattention and peer problems, and associated risk factors for group membership. The sample included 3,578 children (50.8% males) from a population birth cohort in Scotland (Growing Up in Scotland). The parental version of the Strengths and Difficulties Questionnaire (SDQ) was used when children were 4, 5, 6, 7, and 10 years old. Antecedent factors at the perinatal, child, and family levels were collected using parental reports, observation, and standardised assessments at 10, 24, and 36 months. A group-based multi-trajectory analysis was employed. Findings showed that a six-group model best fit the data. Identified groups included non-engagers, normative, decreasing externalising/low peer problems, low externalising/moderate peer problems, moderate externalising/increasing peer problems and multimorbid moderate-high chronic. Findings suggest multimorbidity between externalising behaviours and peer problems in the more elevated groups. Two common protective factors emerged across all groups: caregiver mental health and parent-infant attachment. Identified risk factors were specific to group membership. Risk factors for the most elevated group included single-parent status, social deprivation, previous neonatal intensive care unit admission, child sex, whilst children’s expressive language was a protective factor. Taken together, findings contribute to the emerging literature modelling trajectories of externalising behaviours and peer problems simultaneously and have important practical implications for prevention of problems in childhood, by identifying targets at the perinatal, child, and family levels.

**Supplementary Information:**

The online version contains supplementary material available at 10.1007/s10802-022-00956-8.

## Introduction

Externalising problems, including conduct problems and hyperactivity/inattention, are increasingly common behavioural difficulties, with an estimated ~ 3% of children displaying conduct disorders and ~ 4% presenting with hyperactivity/inattention (Barican et al., 2022; Fairchild et al., 2019). Early externalising problems in childhood have been linked to long-term difficulties such as peer problems and rejection, academic failure, substance abuse, and antisocial behaviours (Bevilacqua et al., 2018; Flouri et al., 2019; Hong et al., 2014; Murray et al., 2021; Pingault et al., 2013). Particular attention has been focused on unpacking the association between externalising behaviours and peer problems during childhood (Haas et al., 2018; Laird et al., 2001). Chen et al., (2015) highlighted both the prospective and concurrent risk association between conduct problems and peer problems such as rejection, while Hoza et al. (2005) demonstrated that children with hyperactivity/inattention score lower on social preference at school and are more likely to be rejected by peers. Reijntjes et al. (2011) conducted a meta-analysis to inspect two prospective paths for this association: the extent to which peer victimisation predicted future changes in externalising problems and whether externalising behaviours predicted later peer problems. They concluded that externalising behaviours are both antecedent and consequences of peer problems across time.

These findings support previous theory describing a bidirectional association between externalising behaviours and peer problems. For example, children exhibiting conduct problems and hyperactivity/inattention difficulties may exasperate other children, subsequently inducing negative reactions from these other children towards them, such as being victimisation or rejection (Thorell et al., 2017). Whereas experiencing negative peer relationships could alternatively lead to difficulties in regulating children’s emotions in adverse social situations, resulting in the appearance of disruptive behaviours (Haltigan & Vaillancourt, 2014; Schwartz et al., 2001) or exhibiting externalising behaviours to defend themselves (Averdijk et al., 2016; Reijntjes et al., 2011). Moreover, the overlap between conduct problems and hyperactivity/inattention has been associated with experiencing more peer problems in childhood, suggesting that negative peer relationships are associated with a range of externalising problems (Andrade & Tannock, 2013). In addition to the long-term consequences of externalising behaviours, peer problems have also been associated with later adverse outcomes, such as poor academic performance, lower self-esteem, and depressive symptoms (Isaacs et al., 2008; Laird et al., 2001). Therefore, considering the long-term negative outcomes of both externalising behaviours and peer problems and the identified association between these difficulties, it is important to gain a better understanding of their longitudinal presentations across childhood and to examine how these behaviours may feed into the other across time. Identification of associated risk and protective factors to understand aetiology of potential concomitant developmental trajectories of externalising problems and peer problems is also warranted.

Longitudinal research has identified multiple trajectory groups when examining individual trajectories of conduct problems, hyperactivity/inattention, and peer problems. For conduct problems, low, moderate-desisting, high-desisting and high-chronic have been described (Olson et al., 2017; Shaw et al., 2003, 2005). In these subgroups, a large majority of children display a normative peak of conduct problems from two to five years old, which then decrease through childhood once cognitive, language, and regulation abilities have been better developed (Cole et al., 2011; Tremblay, 2010). However, a small percentage of children, usually representing ~ 5% of the sampled populations have been found to display persistent conduct problems, which remain high across childhood and adolescence (Girard et al., 2019; Nagin & Tremblay, 1999). In the case of hyperactivity/inattention, studies examining developmental trajectories have suggested a group of children with a general decline in symptoms and another group with stable problems over time (Musser et al., 2016; Sasser et al., 2016; Vergunst et al., 2019). The stability of hyperactivity/inattention has been mainly explained through genetic factors, whereas fluctuations over time are thought to be influenced by environmental determinants (Kan et al., 2013). Finally, some longitudinal studies of peer problems have described subgroups of children with increasing trajectories of peer victimisation, including low/increasing, moderate/increasing, and high/increasing (Barker et al., 2008; Boivin et al., 2010), whereas others have shown a general decline across the school period, with trajectory subtypes identified as low, childhood limited, moderate-emerging stable, and high chronic (Geoffroy et al., 2018; Oncioiu et al., 2020).

Nevertheless, despite increased research on individual trajectories of conduct problems, hyperactivity/inattention, and peer problems, only one study has examined concomitant trajectories of these behaviours across childhood (Girard, 2021). Using a person-centred approach in a European cohort (Ireland; Growing Up in Ireland), Girard (2021) examined the longitudinal presentation of internalising, externalising, and peer problems from three to nine years old, identifying six subgroups who evidenced multimorbidity across behaviours. Within these groups, three presented no or low symptoms, and three showed more elevated problems across time, with the last one displaying persistent multimorbid difficulties (i.e., ‘moderate increasing internalising/moderate decreasing-stable externalising and peer problems’, ‘low increasing internalising/mixed externalising/stable peer problems’, ‘high chronic-increasing multimorbid’). However, as this study inspected joint presentations of internalising, externalising, and peer problems, there is still a need for findings reporting the unique concomitant trajectories of externalising behaviours and peer problems. To the best of our knowledge, no study has simultaneously examined the unique longitudinal presentation of conduct problems, hyperactivity/inattention, and peer problems across childhood.

Furthermore, considering multiple risk *and* protective factors to discriminate between developmental trajectories will provide new understanding around the factors specific to multimorbid conduct problems, hyperactivity/inattention and peer problems. Following the ecological system theory approach (Bronfenbrenner, 1992), perinatal, child, and family risk and protective factors were considered in this study. Perinatal factors such as low birth weight and early experiences in a neonatal unit (NICU) have been associated with higher rates of conduct problems and hyperactivity/inattention (Anderson et al., 2003; Greenley et al., 2007; Mathewson et al., 2017). These factors may reflect neurodevelopmental immaturity (Shum et al., 2008; Skranes et al., 2007) and stressful experiences for the baby in the NICU (Linsell et al., 2019), which may impact later development. At the child level, language abilities and sex have been described as important factors for externalising problems and peer problems outcomes. With respect to children’s language, population-based cohort samples have revealed significant bidirectional associations between language abilities and externalising problems such as conduct problems (Girard et al., 2014, 2016). In addition, the gender paradox theory highlighted that conduct problems and hyperactivity/inattention vary among girls and boys, with boys displaying greater problems over time (Loeber & Keenan, 1994). Proposed reasons for these sex differences may be associated with social and genetic factors. Regarding social factors, it has been suggested that socialisation of gender roles for boys and girls start from early stages in development, with parents and the broader contextual environment, which impact upon distinct childhood problems (Tremblay & Côté, 2019). For example, boys may be socialised to compete and fight, which may increase externalising behaviours. Furthermore, genetic influences have also been studied to clarify sex differences (Dmitrieva et al., 2011), with evidence indicating that girls need more risk genes than boys before exhibiting externalising problems (Rhee & Waldman, 2004; Rhee et al., 1999). Concerning peer problems, there is mixed evidence for sex differences. Some research has suggested that girls are more likely to experience peer problems such as rejection and victimisation (e.g., Humphreys et al., 2013), while others have shown that boys are more likely to experience these types of problems (e.g., Nansel et al., 2001). A potential explanation for these mixed findings is that sex differences may be related to the specific types of peer problems examined. For example, girls may be more likely to experience a higher degree of relational peer problems whereas boys more experience higher levels of physical victimisation (Rose & Rudolph, 2006).

At the family level, several maternal characteristics have been related to children’s poorer outcomes, including young maternal age (Chang et al., 2014), single status (Alavi et al., 2017), lower educational attainment (Alavi et al., 2017), and mental health problems, such as depression (Kingston et al., 2018). Moreover, parent–child attachment style has also been associated with children’s externalising behaviours. For example, meta-analytic data demonstrated that insecure attachments were significantly associated with the development of externalising behaviours (Fearon et al., 2010). Maternal characteristics and interaction styles may cumulatively impact upon children’s development since multiple risk factors, instead of single factors, predict children’s behavioural and social outcomes (Rutter & Sroufe, 2000). Finally, the socioeconomic level (SES) represented by the family’s level of social deprivation has been reported as a significant factor for children’s externalising problems (i.e., conduct problems and attention/hyperactivity; Miller & Votruba-Drzal, 2017; Votruba-Drzal, 2006). Children living in environments characterised by high levels of social deprivation, such as poverty and unemployment, often manifest externalising behaviours, potentially because of higher levels of family stress, less investment in resources such as education and health, and social exclusion (Miller & Votruba-Drzal, 2017).

In summary, externalising behaviours and peer problems have been found to have long term negative consequences for children’s wellbeing (Bevilacqua et al., 2018; Flouri et al., 2019; Hong et al., 2014; Isaacs et al., 2008; Laird et al., 2001; Murray et al., 2021; Pingault et al., 2013). Moreover, associations have been described between externalising behaviours and peer problems (Chen et al., 2015; Healy et al., 2015; Hoza et al., 2005; Keiley et al., 2003; Reijntjes et al., 2011). However, to the best of our knowledge, only one study (Girard, 2021) has inspected multi-trajectories of these behaviours simultaneously, and none to date has focused uniquely on the longitudinal presentations of conduct, hyperactivity/inattention, and peer problems. Consequently, this study has two aims. First, to inspect developmental trajectories of concurrent conduct problems, hyperactivity/inattention, and peer problems in children between four and 10 years of age to identify subgroups following distinct patterns of co- and/or multimorbid problems across childhood. Second, to detect risk and protective factors for group membership. Based on the existing literature (Olson et al., 2017; Shaw et al., 2003, 2005), a four-group model was hypothesised to best fit the data, including a never engaging group with no-to low problems, a normative group with low-moderate decreasing externalising problems and low peer problems, a high-decreasing group with initially high but decreasing externalising problems and moderate peer problems, and a persistent stable high group. We anticipate these different longitudinal presentations since, after the preschool stage, a general decline of externalising problems is expected in the majority of children, with only a small proportion of children exhibiting high stable problems over time. Moreover, according to the reported associations between externalising problems and peer problems, it is also expected that groups with high decreasing or stable conduct problems and hyperactivity/inattention will present with more peer problems across childhood than groups with no to low externalising behaviours. Finally, according to the cumulative risk model (Atzaba-Poria et al., 2004), it was anticipated that these groups would have different aetiological antecedents. More precisely, we anticipated that groups with higher externalising problems and peer problems would present with more associated risk factors at the perinatal (i.e., low birth weight, NICU stay), child (i.e., boys, lower expressive language skills), and family levels (i.e., younger mothers, single parents, no educational qualifications, low mental health scores, low maternal-infant attachment, rural areas, most socially deprived families) than children within the no-engagers or normative groups.

## Methods

### Participants

This study used data from families enrolled in the Growing Up in Scotland (GUS) cohort study. The GUS dataset is a population-based cohort from Scotland launched in 2005 with children identified through the child benefit tax register. The initial sample included two cohorts, a birth cohort (N = 5,217) comprising infants born between 2004 and 2005 and a child cohort (N = 2,858) involving children born between 2002 and 2003. Additional details of recruitment and data collection can be found in Anderson et al. (2007). Data from sweeps 1 to 8 for the birth cohort were used, corresponding to when children were 10 months, two, three, four, five, six, eight and 10 years old. Inclusion criteria in the current study were (i) children who were enrolled in the birth cohort sample to ensure information on antecedents of early risk factors were available, and (ii) children having at least three assessments of conduct, hyperactivity/inattention and peer problems from sweeps 4 to 8, to model quadratic growth. Thus, the final sample in this study includes 3,578 families (68.6% of the total initially recruited sample). Sample characteristics of the included cohort as compared to those excluded from the current study can be found in Table 1. Comparison between samples revealed a greater degree of advantage across birth and demographic characteristics for children included in this study. Ethical approval was granted from Scotland’s Scottish “A” Multicentre Research Ethics Committee (MREC). Informed written parental consent was collected prior to each wave of data collection.

**Table 1:** Demographic characteristics of the excluded sample and the included sample

	Excluded sample (n = 1,639)	Included sample (n = 3,578)	*p*
Low birth weight (yes)	125 (7.7%)	214 (6.0%)	0.024
NICU stay (yes)	221 (13.5%)	383 (10.7%)	0.003
Child sex (male)	788 (52.7%)	1,819 (50.8%)	
Child expressive language	67.9 (20.0)	73.2 (18.2)	≤ 0.001
Maternal age (under 20 years of age)	181 (12.1%)	160 (4.5%)	≤ 0.001
Single parent (yes)	487 (29.7%)	492 (13.8%)	≤ 0.001
Maternal education (no qualifications)	242 (14.8%)	229 (6.4%)	≤ 0.001
Caregiver mental health	0.1 (1.0)	-0.02 (0.9)	0.001
Maternal-infant attachment	24.6 (2.6)	24.2 (2.5)	≤ 0.001
Area (rural)	223 (13.6%)	737 (20.6%)	≤ 0.001
Social deprivation (most deprived)	471 (31.5%)	654 (18.3%)	≤ 0.001

Low birth weight < 2.500 gr. Child expressive language = British Ability Scales version II (BAS-II). Maternal education = having no qualifications as maximum educational level vs. having qualifications (e.g., standard grade, higher grade, vocational qualification, degree). Caregiver mental health = subset of six items from the Depression and Anxiety Stress Scales (DASS). Maternal-infant attachment = subset of six items from the Condon Maternal Postnatal Attachment Scale. Social deprivation = assessed by means of Scottish Index of Multiple Deprivation (SIMD) scores. Most deprived quintile vs. other four quintiles. Means and (standard deviations) are presented for child expressive language and maternal-infant attachment

### Measures

#### Children’s Conduct Problems, Hyperactivity/Inattention, and Peer Problems

Children’s conduct problems, hyperactivity/inattention, and peer problems were assessed using the parent version of the Strengths and Difficulties Questionnaire (SDQ; Goodman, 1997) when children were four, five, six, eight, and 10 years old. The SDQ is a screening tool assessing multiple behavioural and emotional difficulties in children aged three to 16 years old, comprising 25 items classified into five subscales (i.e., emotional symptoms, conduct problems, hyperactivity/inattention, peer problems, and prosocial behaviours). In this study we use the conduct problems, hyperactivity/inattention, and peer problems subscales. Parents rate each item on a 3-point scale from 0 (*not true*) to 2 (*certainly true*), with subscale scores ranging from 0 to 10. According to previously proposed cut-offs (Girard, 2021; YouthInMind, 2013), conduct problems scores were specified as low (0–2), moderate (3), and high (4–10), hyperactivity/inattention problems as low (0–4), moderate (5–6), and high (7–10), and peer problems as low (0–1), moderate (2), and high (3–10). The psychometric properties of the SDQ have been extensively researched and well validated (Goodman, 2001), and the SDQ parent version has demonstrated factorial invariance over time, which supports its use in longitudinal research (Sosu & Schmidt, 2017). Cronbach’s alpha in the current sample were 0.49, 0.66, 0.57, 0.64, 0.60 for the conduct problems subscale, 0.71, 0.79, 0.76, 0.80, 0.79 for the hyperactivity/inattention subscale, and 0.42, 0.52, 0.52, 0.55, 0.55 for the peer problems subscale at four, five, six, eight, and 10 years respectively.

#### Risk and Protective Factors

Antecedent factors were gathered from sweeps 1 to 3, when children were 10, 25, and 36 months, respectively. At sweep 1, parents and fieldworkers provided reports on birth outcomes, child, and family antecedents, including low birth weight (categorised as under 2.500 gr.; yes/no), time in the NICU after childbirth (yes/no), child sex (male/female), maternal age (20 years or less; yes/no), marital status (single/not single), maternal educational level (no educational qualifications; yes/no), area of family residence (urban/rural). Maternal-infant attachment was assessed with maternal reports using a subset of six items from the Condon Maternal Postnatal Attachment Scale (Condon & Corkindale, 1998), with higher scores indicating better attachment with the infant. Cronbach’s alpha in the current sample was 0.55. The social deprivation quintile was measured using Scottish Index of Multiple Deprivation (SIMD) scores, which are assigned according to whether the postcode in which the primary caregiver lives are in the least, second-to-least, middle, second-to-most or most deprived fifths of postcodes overall across Scotland (most deprived; yes/no) (Scottish Government, 2020). At sweep 2, primary caregiver mental health was assessed using the z-scores of a subset of six items from the Depression and Anxiety Stress Scales answered by the primary caregiver (DASS; Lovibond et al., 1995), with positive scores reflecting better than average mental health and negative scores poorer than average mental health. Cronbach’s alpha in the current sample was 0.90. Finally, at sweep 3, children’s expressive language abilities were measured by fieldworkers using the Naming Vocabulary subtest from the British Ability Scales Version 2 (BAS-II; Elliott, Smith, & McCullock, 1997), with higher scores denoting better expressive language abilities. The BAS-II has demonstrated construct validity and correlations with similar measures of cognitive functioning, such as the Wechsler Intelligence Scale for Children (Cook, 1988).

### Statistical Analysis

To examine concomitant trajectories of conduct problems, hyperactivity/inattention, and peer problems, a group-based multi-trajectory modelling analysis (Nagin, 2005; Nagin et al., 2016) was conducted. The group-based multi-trajectory model is an extension to the original group-based modelling analysis (Nagin, 2005) and adheres to a person-centred approach (Magnusson, 2003; Nagin, 2005). This analysis is an application of finite mixture modelling for identifying trajectory groups following diverse developmental patterns and considering multiple variables at once (e.g., conduct problems, hyperactivity/inattention, peer problems). According to Nagin’s recommendations (Nagin, 2005), two steps were followed for group selection: defining the number of groups that best fit the data and selecting the shape of each trajectory by determining polynomial terms for each group. For the first step, two to eight groups were assessed to compare model fit according to the Bayesian Information Criteria (BIC), with a larger BIC (in contrast to alternative modelling approaches) indicating a better fitting model (see Nagin, 2005). The main fit improvement occurred with the six-group model since the following groups were characterised by a more minimal BIC change from group 6 to group 7 and from group 7 to group 8 (Table 2). Further inspection of each model’s graph showed no distinguishing group difference when adding more groups (i.e., 7 and 8 groups). Therefore, considering the main fit improvement of the BIC statistic and the principle of parsimony, a six-group multi-trajectory model was chosen as best describing the distinct characteristics of the data.

**Table 2:** Comparison of the Bayesian Information Criteria (BIC) for assessment of model fit

	BIC	BIC	AIC
2-group	-86319.86 (N = 50370)	-86290.77 (N = 3578)	-86222.77
3-group	-84508.31 (N = 50370)	-84465.99 (N = 3578)	-84367.07
4-group	-83895.64 (N = 50370)	-83840.11 (N = 3578)	-83710.27
5-group	-83404.10 (N = 50,370)	-83335.34 (N = 3578)	-83174.59
**6-group**	**-83089.28 (N = 50370)**	**-83007.30 (N = 3578)**	**-82815.64**
7-group	-82846.49 (N = 50370)	-82751.28 (N = 3578)	-82528.71
8-group	-82667.44 (N = 50370)	-82559.01 (N = 3578)	-82305.52

Sample size in the first BIC column represents the total number of assessments used within the model estimation across time and participants. Sample size in the second BIC column denotes the actual sample size used in the trajectories. The theoretically correct BIC score lies in between these two indices (Nagin, 2005)

For the second step, polynomial terms (i.e., quadratic, linear, and constant) were tested in the six-group multi-trajectory model until each parameter estimate was statistically significant. After selecting the number of groups that best fit the data and selecting the polynomial terms for each group, further criteria, such as the average posterior probabilities and the odds of correct classification were applied to assess the fit of the selected model (Nagin, 2005). The average posterior probabilities denote the chances that an individual displaying a particular pattern of behaviours will be assigned to a specific trajectory group. Odds of correct classification quantifies how much better the model is at allocating participants to groups than randomly assigning participants based on group size. Nagin (2005) suggested that posterior probabilities greater than 0.7 (or 70%) and odds of correct classification higher than 5 indicate good model fit. In this study, both indices offered further support for the six-group multi-trajectory model (Table 3). After model selection, risk factors for group membership were assessed at the bivariate level using analysis of variance and chi-square tests. Subsequently, only statistically significant factors at the bivariate level were entered into the multi-trajectory model estimation. Analyses were conducted using Stata v17.0. The statistical threshold was set to *p* = 0.05. The term significance is used in place of statistical significance henceforward.

**Table 3:** Model fit criterion of conduct problems, attention/hyperactivity and peer problems multi-trajectories

Trajectory Group	*n*	Average Posterior Probability of Group Membership	Odds of Correct Classification
1	761	91.1	37.7
2	593	79.1	19.1
3	661	78.1	15.8
4	520	81.9	26.6
5	742	85.1	21.8
6	301	93.9	169.0

Membership probability greater than 70 and OCC greater than 5 represent good model fit

## Results

### Group-Based Multi-Trajectories

**Fig. 1:**
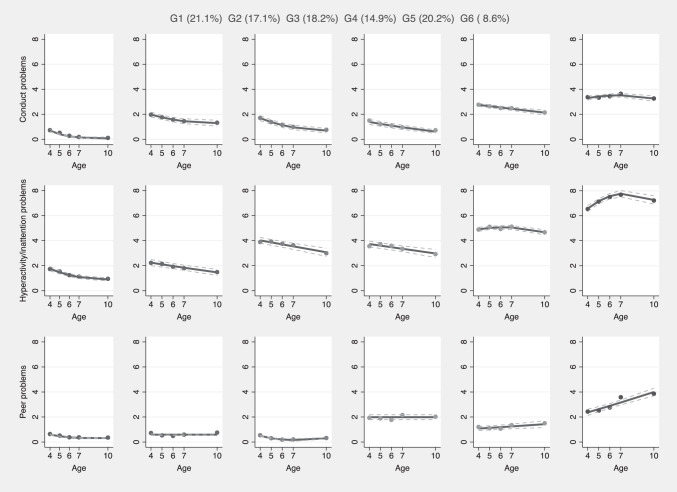
Multi-trajectories of conduct problems, hyperactivity/inattention and peer problems from four to 10 years old

Six trajectory groups were identified, exhibiting distinctive patterns of conduct problems, hyperactivity/inattention, and peer problems from four to 10 years old. Group 1 represented 21.1% of the estimated cohort and was the largest group. This group was labelled ‘non-engagers’ and was characterised by low decreasing conduct problems and hyperactivity/inattention, and low stable peer problems. Group 2 included an estimated 17.1% of children and was labelled ‘normative’. Compared to Group 1, this group also showed low decreasing conduct problems and hyperactivity/inattention, and low stable peer problems. However, the level of both conduct problems and hyperactivity/inattention were more elevated in Group 2 as compared to Group 1. Group 3 comprised an estimated 18.2% of children and was labelled ‘decreasing externalising/low peer problems’. In this group, both conduct and peer problems followed a quadratic trajectory, similar to the first and second groups, showing low decreasing conduct problems and low stable peer problems. Hyperactivity/inattention problems was more elevated in this group as compared to the previous two groups and followed a linear decreasing trajectory between four to ten years old. Group 4 included 14.9% of the estimated cohort and was labelled ‘low externalising/moderate peer problems’. Similar to group 3, conduct problems and hyperactivity/inattention were initially low and followed a decreasing trajectory. In contrast, peer problems were moderate at four years old, remaining constant thereafter. Group 5 included an estimated 20.2% of children and was labelled ‘moderate externalising/increasing peer problems’. Conduct problems were initially moderate and followed a slightly decreasing trajectory, while hyperactivity/inattention started at a moderate level, following a peak at age seven and then marginally decreasing at age 10. Peer problems had a linear increasing trajectory over time, although at a lower initial level and approaching moderate levels at age 10. Finally, Group 6 included an estimated 8.6% of the cohort, labelled as ‘multimorbid moderate-high chronic’. This group was characterised by moderate conduct problems, following a peak at age seven and then slightly decreasing when children were 10 years old. Hyperactivity/inattention problems also followed a quadratic trajectory with a peak at age seven and a marginal decrease by age 10, albeit at a high level across time. Peer problems started at a moderate level and followed an increasing linear trajectory, with high levels of problems from 6 to 10 years old. Multi-trajectory groups are displayed in Fig. 1 and parameters estimates in Supplementary File 1.

### Risk Factors for Group Membership

Bivariate analyses, including analysis of variance and chi-square tests, showed that low birth weight and area of the family dwelling were not significant factors in discriminating between group membership. All the other variables were significant for group membership (see Table 4). Subsequently, all significant factors at the bivariate level were modelled within the multi-trajectory estimation, using the non-engagers trajectory group (Group 1) as the reference group. At the perinatal level, having spent time in the NICU after childbirth increased the likelihood of membership only for the multimorbid moderate-high chronic group (Group 6). At the child level, male sex increased the likelihood of membership in all groups relative to the non-engagers reference group, with the exception of group 2, the normative group. Conversely, children with higher expressive language skills had a decreased likelihood of membership in groups 4, 5 and 6 as compared to the non-engagers group. At the family level, maternal age (i.e., younger mothers, under 20 years of age at childbirth) was not significant in discriminating between group membership. Single mother status increased children’s likelihood of membership in groups 4, 5 and 6. Low maternal educational level (i.e., leaving school with no qualifications) increased the likelihood of group membership only in the moderate externalising/increasing peer problems group (Group 5). Better than average caregiver mental health and higher maternal-infant attachment scores decreased the likelihood of membership in all groups as compared to the non-engagers group. Finally, having a family-of-origin categorised in the most socially deprived quintile (i.e., quintile five) increased the likelihood of membership in the multimorbid moderate-high chronic group (Group 6). Table 5 shows risk factors associated with group membership at the multivariable level.

**Table 4:** Bivariate Analysis of Antecedent Risk Factors by Group

	Group 1	Group 2	Group 3	Group 4	Group 5	Group 6	*p*
*Perinatal Factors*							
Low birth weight (yes)	5.4%	5.4%	5.0%	6.4%	6.3%	9.3%	0.146
NICU stay (yes)	9.1%	9.4%	9.5%	12.1%	10.4%	18.3%	< 0.001
*Child Factors*							
Sex (male)	38.6%	38.8%	57.8%	52.9%	58.0%	69.1%	< 0.001
Expressive language	77.5 (16.4)	76.4 (16.8)	74.9 (15.8)	70.6 (19.2)	69.8 (18.0)	63.2 (23.0)	< 0.001
*Family factors*							
Maternal age (under 20 years of age)	2.1%	3.6%	3.6%	4.4%	6.4%	9.7%	< 0.001
Single parent (yes)	7.6%	8.9%	10.9%	13.7%	19.5%	30.9%	< 0.001
Maternal education (no qualifications)	2.8%	4.6%	4.8%	6.7%	9.7%	14.1%	< 0.001
Caregiver mental health	-0.32 (0.6)	-0.13 (0.7)	-0.17 (0.7)	0.06 (0.9)	0.20 (1.0)	0.56 (1.2)	< 0.001
Maternal-infant attachment	24.8 (2.3)	24.3 (2.5)	24.2 (2.3)	24.1 (2.6)	23.9 (2.5)	23.5 (2.9)	< 0.001
Area (rural)	21.8%	21.8%	22.2%	18.9%	20.4%	15.3%	0.132
Social deprivation (most deprived)	11.2%	15.2%	14.2%	21.4%	23.7%	32.6%	< 0.001

Percentages of prevalence displayed for chi-square analysis; means and (standard deviations) presented in analysis of variance. Group 1 ‘non-engagers’ (*n* = 636), Group 2 ‘normative’ (*n* = 454), Group 3 ‘decreasing externalising/low peer problems’ (*n* = 949), Group 4 ‘low externalising/moderate peer problems’ (*n* = 455), Group 5 ‘moderate externalising/increasing peer problems’ (*n* = 523), and Group 6 ‘multimorbid moderate-high chronic’ (*n* = 302)

**Table 5:** Multivariable Analysis of Risk Factors by Group Membership

	B	SE	*p*	OR
NICU stay (yes)				
Group 2	0.26	0.25	0.297	1.30
Group 3	0.17	0.25	0.504	1.19
Group 4	0.28	0.25	0.255	1.32
Group 5	0.12	0.24	0.634	1.13
Group 6	0.63	0.27	**0.018**	1.90
Child sex (male)				
Group 2	-0.18	0.16	0.254	0.84
Group 3	0.87	0.15	** < 0.001**	2.39
Group 4	0.49	0.16	**0.002**	1.63
Group 5	0.80	0.14	** < 0.001**	2.23
Group 6	1.12	0.19	** < 0.001**	3.10
Child expressive language				
Group 2	-0.01	0.00	0.458	0.99
Group 3	-0.01	0.00	0.096	0.99
Group 4	-0.02	0.00	** < 0.001**	0.98
Group 5	-0.02	0.00	** < 0.001**	0.98
Group 6	-0.04	0.00	** < 0.001**	0.96
Maternal age (under 20 years of age)				
Group 2	0.46	0.56	0.414	1.58
Group 3	0.57	0.49	0.240	1.78
Group 4	0.42	0.50	0.401	1.52
Group 5	0.56	0.45	0.205	1.75
Group 6	0.91	0.47	0.051	2.48
Single parent (yes)				
Group 2	-0.12	0.32	0.715	0.89
Group 3	0.25	0.27	0.359	1.28
Group 4	0.59	0.26	**0.024**	1.80
Group 5	0.76	0.24	**0.002**	2.14
Group 6	1.26	0.27	** < 0.001**	3.53
Maternal education (no qualifications)				
Group 2	0.21	0.43	0.623	1.23
Group 3	0.43	0.39	0.272	1.54
Group 4	0.43	0.39	0.263	1.54
Group 5	0.81	0.34	**0.018**	2.25
Group 6	0.67	0.38	0.083	1.95
Caregiver mental health				
Group 2	-0.63	0.19	**0.001**	0.53
Group 3	-0.41	0.19	**0.026**	0.66
Group 4	-1.18	0.17	** < 0.001**	0.31
Group 5	-1.37	0.16	** < 0.001**	0.25
Group 6	-1.70	0.20	** < 0.001**	0.18
Maternal-infant attachment				
Group 2	-0.10	0.03	**0.001**	0.90
Group 3	-0.11	0.03	** < 0.001**	0.90
Group 4	-0.12	0.03	** < 0.001**	0.89
Group 5	-0.16	0.03	** < 0.001**	0.85
Group 6	-0.25	0.04	** < 0.001**	0.78
Social deprivation (most deprived)				
Group 2	-0.00	0.25	0.995	1.00
Group 3	-0.07	0.24	0.772	0.93
Group 4	0.41	0.22	0.059	1.51
Group 5	0.35	0.20	0.085	1.42
Group 6	0.69	0.23	**0.003**	1.99

The comparison group was Group 1 ‘non-engagers’ (*n* = 636). Group 2 ‘normative’ (*n* = 454), Group 3 ‘decreasing externalising/low peer problems’ (*n* = 949), Group 4 ‘low externalising/moderate peer problems’ (*n* = 455), Group 5 ‘moderate externalising/increasing peer problems’ (*n* = 523), and Group 6 ‘multimorbid moderate-high chronic’ (*n* = 302). Table includes b coefficient, standard error, significant value, and odds ratio

## Discussion

This study contributes new insights to the literature on externalising behaviours and peer problems, having examined concomitant trajectories of conduct problems, hyperactivity/inattention, and peer problems. To the best of our knowledge, this is the first study using a group-based multi-trajectory approach to model these three behaviours simultaneously across childhood and inspect early risk and protective factors associated with group membership. Using the GUS cohort, a nationally representative cohort from Scotland, results showed that a six-group model best fit the data. All six groups evidenced distinctive levels and progression of problems over time. Change across the developmental period examined mainly reflected decreasing and stable trajectories, although increasing trajectories were also evident. In the period of development under investigation, our results suggest multimorbidity between externalising behaviours and peer problems in the more elevated groups over time, with one exception. For group 4, we observed that despite decreasing externalising problems across time, the initially moderate levels of peer problems remained stable. We identified two common protective factors across all groups (i.e., caregiver mental health and maternal-infant attachment), while risk factors identified were specific to group membership. In line with the cumulative risk model, a greater number of risk factors were identified for the more severe problem groups (Group 5 and 6).

### Group-Based Multi-Trajectories

The study’s first aim was to better understand subgroup prevalence and patterns of change within trajectories of conduct problems, hyperactivity/inattention, and peer problems across childhood. Accordingly, a four-group model was hypothesised to best fit the data, including a group with low problems, a normative group with low-moderate decreasing externalising problems and low peer problems, a high-decreasing group, and a high stable group. Findings partially supported the study hypotheses of group selection. Six groups emerged instead of four, with three groups matching our hypotheses (i.e., Groups 1, 2, and 6). The presence of the additional groups, ‘decreasing externalising/low peer problems’ (Group 3), ‘low externalising/moderate peer problems’ (Group 4), and ‘moderate externalising/increasing peer problems’ (Group 5) demonstrated greater developmental heterogeneity than anticipated. Group 3 displayed similar patterns to the first two groups’ conduct and peer problems. However, levels of hyperactivity/inattention in this group, while decreasing, were consistently more elevated across time compared to trajectories for Groups 1 and 2. Group 4 showed similar decreasing trajectories in the externalising problems domains to group 3, whereas peer problems were moderate but remained stable over time. This may reflect the influence of other factors associated with the maintenance of peer problems over time not included in this study, such as emotional problems (Forrest et al., 2020). Moreover, Girard (2021) reported two similar groups to Group 3 and 4 in the current study with respect to the level and progression of both conduct and peer problems over time, albeit with differing progressions of hyperactivity/inattention. In the current study, hyperactivity/inattention decreased over time in both groups whereas they remained stable across time in the former study. These findings suggest that externalising difficulties and peer problems are not occurring in isolation during childhood, and would support previous work demonstrating that behavioural and peer problems feed into the other across time (Hodges & Perry, 1999; Reijntjes et al., 2011; Schwartz et al., 2001).

The presence of multimorbidity in this study has practical and theoretical implications. In a practical sense, children exhibiting multimorbid problems (e.g., groups 5 and 6, combined estimated 28.8%) have been described at a greater risk for later maladaptive outcomes (Newman et al., 1998). Hence, children in the sampled population displaying elevated conduct problems, hyperactivity/inattention and increasing levels of peer problems may develop problematic outcomes in the future, which can potentially carry a higher disease burden and economic costs to societies (Pelham et al., 2007). Therefore, longitudinal identification of multimorbid groups is important for targeting children that may need additional support. From a theoretical perspective, the presence of multimorbidity challenges the classic classification systems of psychopathology, which are characterised by discrete and exclusive problems categories. Thus, multimorbidity findings from this study add evidence for further elaboration of classification systems of behavioural problems (e.g., the hierarchical taxonomy of psychopathology) and developmental psychopathology theories.

As anticipated, this study identified a non-engager (Group 1), normative (Group 2) and high-chronic (multimorbid moderate-high chronic group, Group 6) group. The non-engager and the normative group, a combined estimated 38.2% of children in the sample, exhibited low or declining symptoms in conduct problems, hyperactivity/inattention, and peer problems. These two low risk groups are in line with previous findings in the developmental literature (e.g., Fanti & Henrich, 2010; Girard, 2021; Patalay et al., 2017), suggesting that behavioural problems in preschool children may be normative, particularly as children enter into the classroom setting and are learning to navigate social relationships with peers. For most children, these early difficulties are expected to decline as higher-order skills are better developed, resulting in low-risk trajectories of externalising behaviours and peer problems after entry into formal schooling. Group 3 also followed a low-risk trajectory. Combined, groups 1–3 included 56.4% of children that presented within a normal range of problems in this sample. Finally, as expected, we identified a smaller group of children following a high-risk multimorbid trajectory across all externalising behaviours and peer problems (i.e., the multimorbid moderate-high chronic group), which included an estimated 8.6% of children. Previous developmental studies examining individual and joint trajectories of externalising behaviours and peer problems have also identified smaller groups of children at high-risk for elevated levels of chronically stable and/or increasing problems, however identified groups in these studies have typically consisted of a smaller proportion of the sampled population (i.e., between 3–6%; Shaw et al., 2005; Barker et al., 2008; Girard, 2021; Girard et al., 2019; Nagin & Tremblay, 1999; Patalay et al., 2017). As compared to these other studies, a higher proportion of children in the GUS cohort were identified as belonging to the high-risk group with increasing-chronic problems across multiple domains in childhood. According to previous research in Scotland using the GUS cohort (Marryat et al., 2015; Parkes et at., 2016) and a review of the *Glasgow effect* (Cowley et al., 2016), the higher proportion of children belonging to group 6 may reflect the cumulative impact of multiple life stressors on children’s wellbeing, including deprivation factors (i.e., socioeconomic inequalities, family distress), urban effects (i.e., remote location with lack of social connectedness among neighbours, urban stress), and cultural stressors (i.e., social attitudes).

### Risk and Protective Factors for Group Membership

The second aim of this study was to identify early risk and protective factors for group membership at multiple ecological levels, defining protective factors as influences that change a child’s reaction to environmental hazards that increase the risk of developing problematic outcomes (Rutter, 1985). Two protective factors at the family level were associated with membership in all groups relative to the non-engager group, which included primary caregiver mental health and maternal-infant attachment. Children with mothers reporting better than average mental health were less likely to belong to all groups compared to the non-engagers group, with odds ratio indicating a linear trend across higher problems groups. For example, children were 47%, 34%, 69%, 75% and 82% less likely to belong to groups 2, 3, 4,5 and 6, respectively. These findings are consistent with previous literature indicating that maternal mental health is an important factor in the development of children’s externalising behaviours and peer problems over time (Maruyama et al., 2019). Potential intergenerational transmission of risk mechanisms have been described, including genetic vulnerability, adverse socioeconomic factors, and parenting practices (Elgar et al., 2004). Moreover, maternal-infant attachment was also a protective factor across groups, with an increasing linear trend for groups display higher problems. For each point increase in attachment quality, children were 10%, 10%, 11%, 15% and 22% less likely to belong to groups 2, 3, 4, 5 and 6, respectively. These findings were in line with our expectations given the literature suggesting that caregiver mental health and attachment quality are associated factors that affect mother-infant interactions, and which impact upon children’s later behavioural outcomes (Kim-Cohen et al., 2005). Findings suggest that both protective factors are suitable targets for early prevention efforts, which can be especially beneficial for groups displaying greater problems (i.e., moderate externalising/increasing peer problems and multimorbid moderate-high chronic groups).

Three factors were common for elevated groups and included child sex, receptive language skills, and belonging to single parent households. In line with the literature (e.g., Girard, 2021; Girard et al., 2019; Teymoori et al., 2018), boys were more likely to belong to elevated groups with the largest odds (i.e., 3.1 higher odds) of belonging to the multimorbid moderate-high chronic group. It has been suggested that social determinants for males and females, such as parental and environmental socialisation, impact gender roles behaviours and, in turn, sex differences in childhood problems (Tremblay & Côté, 2019). Consequently, boys have been associated with greater conduct problems and hyperactivity/inattention (Loeber & Keenan, 1994). Genetic influences have also been acknowledged as important for sex differences. For example, evidence from twin studies exhibited a greater genetic influence on hyperactivity/inattention problems in boys but not girls, with girls needing greater liability to manifest symptoms (Rhee & Waldman, 2004; Rhee et al., 1999). Moreover, the gender-environment interaction has also been inspected to explain sex differences in externalising problems, with evidence showing that the social context plays an important role in the gender-specific phenotypic expression of genes (Dmitrieva et al., 2011). Therefore, our study findings add that when conduct problems, hyperactivity/inattention, and peer problems occur together, boys are more likely to belong to groups with greater problems than the non-engagers and normative groups. This result may reflect that boys are more vulnerable to multimorbid childhood problems, as similarly stated in studies modelling joint externalising problems and internalising problems (Fanti & Henrich, 2010; Girard, 2021; Murray et al., 2021). However, further research inspecting the role of social and genetic determinants is needed to better comprehend aetiology and sex differences in multimorbid problems.

In addition, better expressive language abilities at age three were identified as a protective factor for groups 4, 5 and 6. In line with previous research examining associations between children’s early language skills and externalising problems (e.g., Girard et al., 2014, 2016), our results highlight the importance of children’s early language ability to guard against subsequent externalising behaviours and peer problems. When children are better able to express themselves in social situations, the likelihood of using alternative forms of communication such as aggressive or acting out behaviours should decrease. This may consequently help to further reduce the likelihood of peer problems. Furthermore, children with single parents at 10 months old had greater odds (i.e., 1.8, 2.14, and 3.54 higher odds) of belonging to groups 4, 5 and 6, respectively. This finding may reflect the importance of early family support for mothers (Balaji et al., 2007) and its impact on later children’s outcomes. Single mothers may experience greater stress levels for facing parenthood, impacting mental health and mother-infant interactions, and subsequent offspring behavioural problems. However, further work inspecting this factor is necessary before asserting conclusions.

Finally, three factors were specific for group membership in groups 5 and 6. First, children whose mothers had no educational qualifications had an increased odds more than two-fold for group membership in group 5. Previous studies have reported the importance of maternal education in children’s behavioural outcomes independent of their socioeconomic status (Kalff et al., 2001). This association may be explained via shaping parenting practices related to having more knowledge and experience in the educational system. Surprisingly, maternal education was not similarly a risk factor for the multimorbid moderate-high chronic group (group 6), warranting further investigation. Two factors were significantly associated with increased likelihood of membership in group 6: NICU admission and social deprivation. Children who spent time in the NICU after birth had an increased odds of 1.9 for membership in the multimorbid moderate-high chronic group. Potential mechanisms suggested for the association between having stayed in the NICU and later externalising problems have included the stressful environment of the NICU, early experiences to invasive interventions, and the limited early interactions with caregivers, which may consequently reduce early stimulation and impact upon later social competencies (Linsell et al., 2019). Regarding social deprivation, children from families belonging to the most deprived quintile had two-fold odds of membership in the multimorbid moderate-high chronic group. Potential pathways to explain this association include the family stress model, which suggests that socioeconomic deprivation impacts parental mental health through economic stress, affecting parent–child interactions and later children’s outcomes (Elder, 1977).

Overall, the multimorbid moderate-high chronic group (Group 6) had more associated risk factors for group membership, followed by group 5 with some overlaps. This finding was expected since, according to the cumulative model (Atzaba-Poria et al., 2004; Rutter & Sroufe, 2000), groups with higher problems (i.e., groups 5 and 6) would have more associated risk factors than children within the no-engagers or normative groups. For these two groups, being a boy, staying in the NICU, displaying less developed language skills, having a single parent with no educational qualifications and with socioeconomic deprivation, cumulatively increases the risk for greater and more stable externalising problems and peer problems. In contrast, children with mothers reporting better than average mental health and attachment quality are less likely to develop these difficulties across childhood. All these risks *and* protective factors are already evident early in infancy and toddlerhood, suggesting clear implications for public health via social determinants of mental health and informing targets for prevention. For example, selective prevention strategies suggested by the *National Institute for Health and Care Excellence* (NICE) included the following risk factors for externalising problems: low socioeconomic status, low school achievement, child abuse or parental conflict, separated parents, parental mental health or substance abuse, and parental contact with the criminal justice system (NICE, 2017). However, important antecedent factors identified in this study could complement the ones suggested by NICE, including postnatal health antecedents (i.e., NICU stay), child sex (i.e., boys), and children with lower expressive language skills. Regarding early intervention strategies, NICE guidelines recommend interventions based on social learning theory for conduct problems in children aged three to 11 years old (NICE, 2017) and parenting training programmes for hyperactivity/inattention difficulties in children aged five years or younger (NICE, 2018). Considering the importance of attachment quality and parents’ mental health as protective factors within the context of the current findings, we also recommend exploring hybrid parenting interventions based on cognitive and attachment theory such as Mellow Parenting (Levi et al., 2019) or social learning theory and attachment theory like the Incredible Years (Webster-Stratton, 2001). Furthermore, in light of the multimorbidity between externalising behaviours and peer problems in the elevated symptoms groups, prevention and intervention efforts should also evaluate and incorporate strategies for addressing negative peer relationships that may precede or emerge alongside or as a consequence of externalising problems.

### Strengths and Limitations

This study has several strengths, including being the first one using a group-based multi-trajectory approach to model conduct problems, hyperactivity/inattention and peer problems simultaneously across childhood, whilst using a large and representative population-based cohort from Scotland. In addition, we inspected several variables at multiple ecological levels as predictors for group membership, while using a multi-informant approach. Nonetheless, our findings are subject to some limitations to consider. First, the parent-report version of the SDQ was utilised to assess children’s outcomes, relying only on maternal reports. The combination of both maternal and teacher-reported SDQ would have been optimal considering that peer problems are generally detected at school. Nevertheless, maternal reports are frequently used in developmental research, and the parent-report of the SDQ has robust validity (Stone et al., 2010). Second, even when psychometric properties of the SDQ have been well validated (Goodman, 2001; Stone et al., 2010), for this study, Cronbach’s alpha was lower than the desirable threshold of ≥ 0.70 (Nunnally, 1978) in the conduct and peer problems scales. Moreover, the Condon Maternal Postnatal Attachment Scale also presented a Cronbach’s alpha below this threshold. A potential effect of this is the underestimation of the conduct and peer problems trajectories and their associated predictors – including maternal-infant attachment – because attenuation is more likely due to the diminution of maximal observable associations (Schmitt, 1996). Third, in order to properly apply quadratic growth, we only included children with at least three assessments in the period under investigation, reducing the final sample size used in this study. Attrition between the GUS birth cohort and the included sample exhibited several demographic disparities that may impact population representation. Comparison between samples revealed an underrepresentation of children with postnatal medical antecedents, younger mothers with lower educational levels, and socioeconomically deprived families, among other variables. Therefore, considering the underrepresentation of families facing more risk factors at the perinatal, child and family levels, it is possible that our findings may be specific to the subsample used in this study, requiring future replication to generalise results. Fourth, even though the inspection of risk and protective factors at the perinatal, child, and family levels provided further insights about each trajectory groups antecedent factors that were associated with group-membership, there are still ambiguities about the causal aetiology of co- and multi-comorbid trajectories of conduct problems, hyperactivity/inattention, and peer problems. Consequently, future research inspecting underlying causal mechanisms is needed to better understand the aetiology and developmental pathways of these problems. Finally, the GUS birth cohort data was only available until children were 10 years old. Thus, it was not possible to examine the progression of these developmental trajectories into adolescence, which is an important developmental period when changes in externalising behaviours and peer problems may become apparent.

## Conclusions

Using a person-centred approach, this study reported a group-based multi-trajectory modelling analysis of conduct problems, hyperactivity/inattention, and peer problems using a large population cohort of Scottish families (GUS). Findings showed that six groups best capture the distinct features in the data. Identified groups included children within a normal range of problems (56.4%), children with moderate externalising problems or/and peer problems (35%) and a group with high increasing/stable difficulties over time (8.6%). The more elevated groups suggest multimorbidity between externalising behaviours and peer problems. In addition, risk factors for group membership were identified, suggesting early targets for prevention and intervention programmes for children with the most elevated conduct problems, hyperactivity/inattention, and peer problems. For example, at the child level, prevention strategies should target families with boys who stayed in NICU after childbirth and have lower language skills. At the family level, families with single parents, with poorer maternal mental health and lower levels of mother-infant attachment levels, and who are in the most deprived quintile should also be targeted by prevention strategies. Furthermore, early parenting intervention programmes (e.g., Mellow Parenting, Incredible Years) may increase protective factors such as attachment quality and parents’ mental health, helping to counterbalance the effects of early risk factors. Directions for future research to build upon the current work should include a focus on understanding casual mechanisms, particularly related to the higher risk groups, in an effort to better inform prevention efforts’.

## Supplementary Information

Below is the link to the electronic supplementary material.

Supplementary file1 (DOCX 25.6 KB)

## Data Availability

The Growing Up in Scotland data that support the conclusions of this article are available in the UK Data Service repository, https://beta.ukdataservice.ac.uk/datacatalogue/.
