# Supplementary material for: Multi-Trajectories of Conduct Problems, Hyperactivity/Inattention, and Peer Problems Across Childhood: Results from the Growing Up in Scotland Birth Cohort
Source: Res Child Adolesc Psychopathol. 2022 Aug 3;51(1):55–69. doi: 10.1007/s10802-022-00956-8 (PMC9763152; doi:10.1007/s10802-022-00956-8)
Supplement: Supplementary file 1 — Supplementary file1 (DOCX 25.6 KB) [file 10802_2022_956_MOESM1_ESM.docx]

**Supplementary file I: Multi-Trajectory Parameter Estimates**

Table S1: Multi-Trajectory Parameter Estimates for the 6-Group Model

| Group | Parameter | Estimate | SE | T | *p* |
| --- | --- | --- | --- | --- | --- |
| Conduct problems |  |  |  |  |  |
| 1 | Intercept | 3.74 | 0.39 | 9.51 | 0.000 |
|  | Linear | -1.08 | 0.13 | -8.64 | 0.000 |
|  | Quadratic | 0.05 | 0.01 | 5.86 | 0.000 |
| 2 | Intercept | 3.38 | 0.37 | 9.26 | 0.000 |
|  | Linear | -0.45 | 0.11 | -4.08 | 0.000 |
|  | Quadratic | 0.02 | 0.01 | 2.96 | 0.003 |
| 3 | Intercept | 3.70 | 0.36 | 10.26 | 0.000 |
|  | Linear | -0.64 | 0.11 | -5.77 | 0.000 |
|  | Quadratic | 0.03 | 0.01 | 3.84 | 0.000 |
| 4 | Intercept | 2.00 | 0.13 | 15.85 | 0.000 |
|  | Linear | -0.19 | 0.01 | -9.91 | 0.000 |
| 5 | Intercept | 3.18 | 0.09 | 34.15 | 0.000 |
|  | Linear | -0.11 | 0.01 | -7.81 | 0.000 |
| 6 | Intercept | 2.27 | 0.48 | 4.71 | 0.000 |
|  | Linear | 0.36 | 0.15 | 2.48 | 0.013 |
|  | Quadratic | -0.03 | 0.01 | -2.56 | 0.010 |
| Hyperactivity/inattention |  |  |  |  |  |
| 1 | Intercept | 3.69 | 0.39 | 9.49 | 0.000 |
|  | Linear | -0.66 | 0.12 | -5.50 | 0.000 |
|  | Quadratic | 0.03 | 0.01 | 3.88 | 0.000 |
| 2 | Intercept | 2.78 | 0.15 | 18.23 | 0.000 |
|  | Linear | -0.15 | 0.02 | -7.79 | 0.000 |
| 3 | Intercept | 4.67 | 0.15 | 31.17 | 0.000 |
|  | Linear | -0.16 | 0.02 | -8.31 | 0.000 |
| 4 | Intercept | 4.24 | 0.16 | 26.46 | 0.000 |
|  | Linear | -0.13 | 0.02 | -5.95 | 0.000 |
| 5 | Intercept | 3.89 | 0.41 | 9.42 | 0.000 |
|  | Linear | 0.37 | 0.13 | 2.97 | 0.001 |
|  | Quadratic | -0.03 | 0.01 | -3.38 | 0.001 |
| 6 | Intercept | 2.11 | 0.62 | 3.41 | 0.001 |
|  | Linear | 1.52 | 0.19 | 8.01 | 0.000 |
|  | Quadratic | -0.10 | 0.01 | -7.48 | 0.000 |
| Peer problems |  |  |  |  |  |
| 1 | Intercept | 1.75 | 0.53 | 3.32 | 0.001 |
|  | Linear | -0.70 | 0.16 | -4.35 | 0.000 |
|  | Quadratic | 0.04 | 0.01 | 3.53 | 0.000 |
| 2 | Intercept | -0.44 | 0.12 | -3.75 | 0.000 |
| 3 | Intercept | 4.26 | 0.72 | 5.89 | 0.000 |
|  | Linear | -1.67 | 0.23 | -7.27 | 0.000 |
|  | Quadratic | 0.11 | 0.02 | 6.90 | 0.000 |
| 4 | Intercept | 1.79 | 0.08 | 22.93 | 0.000 |
| 5 | Intercept | 0.17 | 0.15 | 1.14 | 0.256 |
|  | Linear | 0.09 | 0.02 | 4.28 | 0.000 |
| 6 | Intercept | 1.09 | 0.19 | 5.73 | 0.000 |
|  | Linear | 0.29 | 0.03 | 10.09 | 0.000 |

Note: Group 1 ‘non-engagers’ (n = 636), Group 2 ‘normative’ (n = 454), Group 3 ‘decreasing externalising/low peer problems’ (n = 949), Group 4 ‘low externalising/moderate peer problems’ (n = 455), Group 5 ‘moderate externalising/increasing peer problems’ (n = 523), and Group 6 ‘multimorbid moderate-high chronic’ (n = 302).
